# Objectively measured physical activity among treatment seeking children and adolescents with severe obesity and normal weight peers

**DOI:** 10.1002/osp4.624

**Published:** 2022-06-13

**Authors:** Yngvild S. Danielsen, Hanna F. Skjåkødegård, Marit Mongstad, Sigurd W. Hystad, Sven J. G. Olsson, Malin Kleppe, Petur B. Juliusson, Bente Frisk

**Affiliations:** ^1^ Department of Clinical Psychology University of Bergen Bergen Norway; ^2^ Department of Clinical Science University of Bergen Bergen Norway; ^3^ Department of Health and Functioning Western Norway University of Applied Sciences Bergen Norway; ^4^ Department of Psychosocial Science University of Bergen Bergen Norway; ^5^ Independent Researcher Stockholm Sweden; ^6^ Department of Medicine Haukeland University Hospital Bergen Norway; ^7^ Children and Youth Clinic Haukeland University Hospital Bergen Norway; ^8^ Department of Health Registry Research and Development Norwegian Institute of Public Health Bergen Norway

**Keywords:** accelerometer, childhood obesity, physical activity, screen time, sleep

## Abstract

**Background:**

Treatment seeking children and adolescents with severe obesity often experience barriers to physical activity. Studies objectively measuring physical activity in this group and investigating explanatory factors for physical activity levels could inform clinical practice.

**Objectives:**

This study aimed to compare objectively measured physical activity levels among treatment seeking children and adolescents with severe obesity and normal weight peers, and to investigate explanatory factors for time spent in moderate physical activity and vigorous physical activity among children and adolescents with severe obesity.

**Methods:**

Children with severe obesity (n = 85) were matched 1:1 by age, gender, and the season for accelerometer measurements with normal weight peers (n = 85). Children wore accelerometers for seven consecutive days, yielding measures of physical activity, sleep duration and timing. Parents reported on screen time, parental body mass index and participation in organized sports.

**Results:**

Children and adolescents with severe obesity spent significantly less time in moderate physical activity (12 min, *p* < 0.001) and vigorous physical activity (21 min, *p* < 0.001) per day compared to normal weight peers. No difference for time spent in sedentary activity was found between groups. For participants with severe obesity, age ≤12 years (*p* = 0.009) and participation in organized sports (*p* = 0.023) were related to more moderate physical activity, while age ≤12 years (*p* = 0.038) and early sleep timing (*p* = 0.019) were related to more vigorous physical activity.

**Conclusion:**

Children and adolescents with severe obesity were less physically active than their normal weight peers. Factors related to more moderate and vigorous physical activity in children with severe obesity were lower age, participation in organized sports and earlier sleep timing.

## 1 INTRODUCTION

Modification of physical activity behavior is a key component in lifestyle interventions for childhood obesity.^1^ However, sustainable, healthy changes in activity levels are seldom achieved.^2^ Increased understanding of physical activity behavior and explanatory factors for activity levels in treatment seeking children with severe obesity is an important next step to improve the physical activity component in lifestyle treatment programs currently delivered to this patient group.

National guidelines for physical activity commonly recommend 60 min of moderate‐to‐vigorous (MVPA) physical activity as a daily minimum for school‐aged children and adolescents.^3^ In a study of Norwegian 9‐year‐old children, 72.1% of the girls and 89.2% of the boys with normal weight reached these recommendations, while for children with overweight the numbers were 61.0% and 70.2, respectively.^4^ These numbers are in agreement with a majority of previous studies concluding that children with obesity are less physically active than normal weight children.^5^, ^6^, ^7^ In addition, emerging differences in MVPA from 6 years of age to middle adolescence (ages 14–17) are reported.^8^ However, studies reporting on differences in physical activity levels amongst children with obesity and normal weight are mainly based on community samples, with few studies conducted with a clinical sample of children and adolescents with severe obesity.^9^ This treatment seeking group of children may have more and other barriers for physical activity than children with less severe forms of obesity. Further, more studies using objective measures of physical activity are still needed, as self‐reported data on activity levels often are found to overreport time spent in MVPA.^10^

Explanatory factors for the identified difference in physical activity levels between children with normal weight and obesity are not fully understood. Several explanatory factors for time spent in MVPA for children across all weight categories have been put forward during the last decades. Level of physical activity have often been investigated in relationship to screen time and sleep duration, with mixed findings.^11^, ^12^, ^13^, ^14^, ^15^, ^16^ Some studies find a relationship between low levels of physical activity and more screen time,^14^, ^15^ while others find no association.^16^ For sleep duration the findings are similar, with some studies reporting an association between short sleep duration and decreased physical activity levels and others no association.^11^, ^12^, ^13^, ^17^, ^18^, ^19^ Interestingly, later timing of sleep has recently been suggested as more strongly linked to lower physical activity levels in children and adolescents than sleep duration.^20^, ^21^ Olds et al.^22^ found late sleep and rise time in adolescents to be associated with more screen time, less MVPA and increased obesity independent of sleep duration. Another suggested explanatory factor for time spent in physical activity is participation in sports,^23^, ^24^ with children engaged in organized sports being more physical active.^23^ Nevertheless, more investigation of behavioral, environmental, interpersonal and individual factors for time spent in physical activity among children and adolescents with obesity is needed. This could be particularly informative for clinical practice related to children with severe obesity that often report more barriers to physical activity.^5^

The aim of the present study was therefore to compare objectively measured physical activity levels among treatment seeking children and adolescent with severe obesity and a matched group of normal weight peers. Further, to investigate explanatory factors (age, sex, sleep timing and duration, screen time, parental body mass index (BMI; kg/m^2^) and participation in organized sports), for time spent in moderate physical activity (MPA) and vigorous physical activity (VPA) among children and adolescent with severe obesity. The hypotheses were that children and adolescents with severe obesity spent less time in physical activity compared to normal weight children and adolescents, and that increased age, female gender, more screen time, as well as shorter sleep and later sleep timing would be associated with less MPA and VPA.

## 2 METHODS

### 2.1 Study design and participants

In this case‐control study 170 (100 girls) children and adolescents between 5.8 and 17.1 years were included. Participants aged ≤12 years were defined as children (n = 78) and participants aged >12 years as adolescents (n = 92). Eighty‐five children and adolescents with severe obesity participated prior to attending a family‐based behavioral treatment of childhood and obesity (FABO) program.^25^ The FABO‐study was conducted at a specialist department for treatment of severe pediatric obesity at the Obesity Outpatient Clinic, Haukeland University Hospital, Bergen, Norway. The department has catchment area responsibilities for all children and adolescents with severe obesity that are entitled to publicly financed treatment. The inclusion criteria were BMI above the International Obesity Task Force (IOTF) cut‐off ≥ 35, or BMI ≥ IOTF 30^26^ with obesity related comorbidity (e.g., psychosocial problems or emergence of cardio‐metabolic risk factors).^25^ Participants were excluded if they were enrolled in any other treatment targeting weight reduction or had severe somatic or psychiatric illness that could affect full participation in the FABO program. All referred children fitting these criteria and living within an hour drive from the hospital were consecutively invited to participate in the FABO‐study and the 85 first were included in this study.

The comparison group consisted of 85 children and adolescents with normal weight (BMI ≤ IOTF 25) recruited from randomly selected schools in Bergen municipality. Stratified random sampling ensured that the obesity and normal weight group were matched 1:1 on age, sex, and season for data collection (April–September vs. October–March). Seasonal matching was conducted to prevent bias in the comparison on physical activity data, due to large seasonal differences in weather and hours of daylight in Norway. Data were collected over a period of 4 years (2014–2018).

Participation in the study was voluntary. An informed written consent was obtained prior to inclusion in the study. For the participating children with obesity, other possible treatment programs were discussed before inclusion. The study was approved by the Norwegian Regional Committee for Medical and Health Research Ethics (2013/1300) and the treatment study registered at http://clinicaltrials.gov (NCT02687516).

### 2.2 Measures

For the group of children and adolescents with obesity, baseline measurements from the FABO‐study were used in the analyses.^25^ The primary outcome in the current study was accelerometer measured physical activity, and included explanatory factors were anthropometric measures, sleep measures, screen time and participation in organized sports and demographic information.^25^

### 2.3 Demographic information

The parents/carers self‐reported on a questionnaire about demographic information, which included information about family structure, parental education level and weight status, child participation in organized sports and daily screen time. The questions “Are both parents living together?” and “Does the child live together with siblings?” were used to evaluate family structure. Parental education level was categorized as either low (≤3 years after secondary school) or high (>4 years after secondary school). Participation in organized sports was reported as “1 = yes” or “2 = no” and daily screen time was rated on a scale (1 = never, 2 = less than 30 min, 3 = ½–1 h, 4 = 2–3 h, 5 = 3–4 h, 6=>4 h of screen time). The questionnaire was completed at the Obesity Outpatient Clinic by the parents/carers of the participants with severe obesity and sent/and returned by mail to the parents of the normal weight participants.

### 2.4 Anthropometric measures

Height and weight were measured by trained personnel at the Obesity Outpatient clinic for the children with obesity and by a trained physiotherapist at the school nurse's office for the normal weight children. The participants were wearing light indoor clothing and no shoes during measurements. For the children and adolescents with severe obesity, InBody 720 (Biospace) was used to measure weight to the nearest 0.1 kg and height was measured by using a wall‐mounted electronic stadiometer (Seca 264, Seca) to the nearest 0.1 cm. For the normal weight children and adolescents, Harpeden portable stadiometer (Crosswell) was used to measure height to the nearest 0.1 cm and weight was measured using a calibrated Seca persona digital scale (Hamburg, Germany) to the nearest 0.1 kg. BMI was calculated using the formula kg/m^2^ and converted to standard deviation scores (SDS) by adjusting for age and sex^27^ using Norwegian growth references.^28^

### 2.5 Physical activity measures

Physical activity was measured using Actiwatch 2 (Philips, Respironics, BEND, OR) for seven consecutive days. Actiwatch 2 is a wrist‐worn accelerometer that records all uni‐axial movement over 0.05 G in periods of 30 seconds. The device was worn on the non‐dominant hand and the registrations were retrieved between 8 AM and 9 PM. Wrist‐worn accelerometers are validated for measuring physical activity in children,^29^ and were chosen to maximize compliance.^30^ Activity data was downloaded using Respironics Actiware software version 6.0.9 and transferred to Microsoft Excel 2016.

Metabolic equivalent of task (MET) was chosen to define intensity level due to its qualities of predicting energy expenditure across body composition. All activity data was reported as counts per 30 s and further divided into different activity levels using a tailored‐made algorithm and transformed into MET. According to Ekblom et al.^27^ cut‐off ranges of 0–160 counts/30 s, 161–524 counts/30 s, 525–811 counts/30 s and >812 counts/30 s corresponds to <1.5 MET (sedentary), 1.5–3 MET (light physical activity (LPA)), 3‐6 MET (MPA) and >6 MET (VPA). To be included in the analysis, the participants needed at least 10 h of wear with Actiwatch 2 each day and a minimum of 4 days of recorded data. Physical activity level was operationalized as mean minutes in LPA, MPA, VPA, MVPA and sedentary activity per day.

### 2.6 Sleep measures

Sleep pattern was objectively measured using Actiwatch 2 (Philips, Respironics, BEND, OR). Wrist‐worn accelerometers have been validated for measuring sleep in children.^31^, ^32^ As for physical activity, data was collected in 30 seconds epochs. Using a medium sensitivity threshold, the epochs were sorted as either “wake” or “sleep”. This threshold was chosen as it has proven to be the least biased estimates of wakefulness, total sleep time and wake after sleep onset in school‐aged children.^33^ To ensure correct sleep measurements, the participants were instructed to press the event marker when turning of light at night and when waking up in the morning. To be included in the analysis, data for at least 5 days, including at least three school nights and two weekend nights, had to be recorded.

Time spent in bed was defined as the rest interval. Sleep time within the main rest interval was further detected by a standard default algorithm in the Respironics Actiware software version 6.0.9. To yield more precise rest intervals, data was also manually scored according to a standardized scoring protocol.^27^ Two independent observers scored 30.0% of the recordings twice, to ensure inter‐rater reliability. The scoring was compared on *total time in bed* and *total sleep time*, and had a 99.6% and 99.9% agreement, respectively. Sleep timing was operationalized as mid‐sleep time defined by the formula: (sleep onset time + sleep offset time)/2. Mid‐sleep time is the midpoint between time for sleep onset and wake up time (sleep offset). If you go to bed at 10 PM and get up at 10 AM, midpoint sleep is estimated to 4 AM. The variable for mid‐sleep time included in the analyses is a calculation of a daily average of 5–7 wear days.

### 2.7 Statistics

Descriptive statistics (mean, percentage, and standard deviation (SD)) was used to describe the study population, including physical activity and sleep measures. Comparison of continuous variables across groups was analyzed by using independent sample *t*‐tests and categorical variables by using chi‐square tests. Cohens *d* effect sizes for group differences on continuous variables were calculated. An effect size of 0.2 is considered a small, 0.5 a medium effect and 0.8 a large effect size.^34^ The relationship between demographic data, anthropometric measures, physical activity, and sleep measures in the obesity group was investigated by using Pearson product‐moment correlation coefficients. The relationship between 3‐6 MET (MPA) and >6 MET (VPA), respectively, amongst the group of children with obesity, and possible explanatory variables were analyzed by unadjusted and adjusted linear regression analyses. Investigated variables were, age, sex, parents BMI and education level, total sleep time, midpoint of sleep, screen time and participation in organized sports. For the adjusted regression analyses, variables were chosen based on their plausible influence on the dependent variable. This included demographic data (age over/under 12 years and sex), as well as sleep measures, leisure time activities (screen time and organized sports) and mother's BMI.

Estimated regression coefficients are presented with 95% confidence interval (CI) and *p*‐values. The significance level was set to 0.05. All statistical analyses were performed with SPSS Statistics 26 (SPSS Inc.) after being controlled for prerequisites needed to run these analyses.

### 2.8 Power estimates

To determine required sample size, G*Power version 3.1.3 was used. The statistical significance was determined by *α* = 0.05 (two‐tailed) and power (1‐β) of 0.8 (80%). To detect a medium effect size (Cohen's *d* = 0.5) for the group comparisons, 64 individuals in both groups was required. Eighty‐five children and adolescents were recruited to each group as this was the sample size recruited to the FABO‐study at the time the matched comparison group was collected.

### 2.9 Missing data

Out of 170 included participants, 154 children and adolescents (90.5%) provided valid accelerometer measured physical activity data and were included in the activity analyses. In total 16 (9.4%) of the participants (12 children and adolescents with severe obesity) were excluded from the activity analysis due to less than 10 h of wear time between 8 AM–9 PM and/or less than 4 days of recordings. For the sleep measurements, 168 (98.8%) participants provided valid accelerometer data. The questionnaire of demographic data was completed by all the parents (100%) of the children and adolescents with severe obesity, and by 65 (76.5%) of the parents of the normal weight children and adolescents. Missing data were not imputed, and pair wise deletion was used in the analyses.

## 3 RESULTS

Characteristics of the participants (n = 170) are presented in Table 1. Summarized, the participants' mean age was 12 ± 3 years, ranging from 5.9 to 17.1 years in the group of children with obesity and from 5.8 to 16.4 years in the group with normal weight peers. The children and adolescents with severe obesity participated in less organized sports (31.8% vs. 69.4%, *p* < 0.001) and had more screen time (4 h per day or more) (43.5% vs. 17.7%, *p* = 0.003), respectively, compared to the normal weight peers. The normal weight children and adolescents were significantly more likely to live with siblings (95.5% vs. 69.4%, *p* < 0.001) and their biological parents (89.4% vs. 56.5%, *p* < 0.001), as well as having parents with a higher education level (mother *p* < 0.001, father *p* = 0.003) compared to the children and adolescents with severe obesity.

**TABLE 1:** Characteristics of the participants

	Obesity n = 85	Normal weight *N* = 85	*p*‐value
Age (mean ± SD, range)	12.1 ± 2.9	12.0 ± 2.8	NS
	5.9–17.1	5.8–16.4	
Sex boys/girls (%girls)	35/50 (58.8%)	35/50 (58.8%)	
BMI	31.3	18.0	<0.001
BMI SDS (mean ± SD)	2.9 ± 0.5	−0.3 ± 0.8	<0.001
*Parent reported data*			
Respons rate (%)	85 (100.0%)	65 (77.0%)	
BMI mother (mean ± SD)	29.9 ± 5.1	NA	
BMI father (mean ± SD)	30.0 ± 5.4	NA	
Mother, completed education (%)			
Elementary school	11.9%	3.0%	
High school	46.4%	28.8%	
College/university <4 years	20.2%	34.8%	
College/university >4 years	20.2%	33.3%	<0.001
Father, completed education (%)			
Elementary school	20.5%	0.0%	
High school	41.0%	45.4%	
College/university <4 years	19.2%	29.7%	
College/university >4 years	11.5%	23.4%	0.003
Biological parents living together (%)	56.5%	89.4%	<0.001
Living with siblings (%)	69.4%	95.5%	<0.001
Children participating in organized sports (%)	32%	69%	<0.001
Children's daily screen time, hours (%)			
0–1 h/day	24.8%	18.8%	
2–3 h/day	30.6%	41.2%	
4 h	12.9%	11.8%	
> 4 h	30.6%	5.9%	0.003

Abbreviations: BMI = kg/m^2^; BMI, body mass index; BMI SDS, body mass index z‐scores; NA, not applicable; SD, standard deviation.

### 3.1 Physical activity and sleep measurements in children and adolescent with obesity and normal weight

**FIGURE 1:**
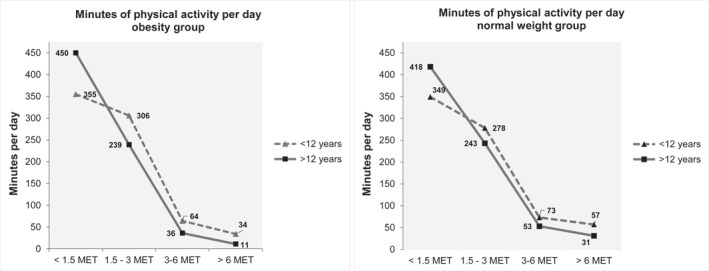
Physical activity across age in children and adolescent with obesity versus children and adolescent with normal weight. < 1.5 MET is defined as sedentary activities, 1.5 – 3 MET as LPA, 3‐6 MET as MPA and >6 MET as VPA. Children = **≤**12 years, adolescents = >12 years

Physical activity and sleep measurements are presented in Table 2. The normal weight children and adolescents spent more time in MVPA compared to the children and adolescent with severe obesity: 105 (range 203.3) versus 72 min (range 172.8) per day (*p* < 0.001). Percentage of children reaching the national recommendations of 60 min^3^ MVPA per day were 60.7% for the group of children with obesity and 85.2% for the children with normal weight, respectively (*p* < 0.001). No statistical differences were found between the groups for light or sedentary activity (<3 MET). The normal weight children and adolescents had earlier sleep timing than the children and adolescents with severe obesity (*p* < 0.001), but there was no statistical difference in total sleep time. Figure 1 presents differences in physical activity levels for the children with obesity and the normal weight group across age: children (≤12 years) and adolescent (>12 years).

**TABLE 2:** Physical activity data and sleep measurements recorded from ActiWatch 2

	Obesity (n = 73)	Normal weight (n = 81)	*p*‐value	Cohens *d*
Number of days with valid measurements (mean ± SD)	6.0 ± 0.8	6.6 ± 0.6	0.280	
*Physical activity*				
Mean numbers of minutes per day (mean ± SD)				
**Sedentary (< 1.5 MET)**	403 ± 79	386 ± 72	0.160	0.2
**LPA (1.5–3 MET)**	272 ± 56	259 ± 44	0.120	0.3
**MPA (3–6 MET)**	50 ± 24	62 ± 21	**<0.001**	0.5
**VPA (> 6 MET)**	22 ± 18	43 ± 26	**<0.001**	0.9
**Low PA (< 3MET)**	344 ± 88	365 ± 75	0.110	0.3
**MVPA (> 3 MET)**	72 ± 42	105 ± 47	**<0.001**	0.7
*Sleep measurements*	7.8 ± 0.8	7.9 ± 0.7	0.608	0.1
Total sleep time, hours (mean ± SD)				
Mid sleep time, time point (mean ± SD)	03:35 ± 1.1	03:02 ± 0.5	**< 0.001**	0.4

*Note*: ActiWatch data processed with the cut offs from Ekblom et al. (2012). Mid sleep time is presented as a time point.

Abbreviations: LPA, light physical activity; MET, Metabolic equivalent of task; MPA, moderate physical activity; SD, standard deviation; VPA, vigorous physical activity. *p*‐values < 0.05 are marked in bold.

### 3.2 Physical activity levels and explanatory factors for children and adolescents with severe obesity

The relationship between MPA, VPA and explanatory variables are presented in Table 3.

**TABLE 3:** The relationship between physical activity and explanatory factors for children and adolescents with severe obesity

	Unadjusted		Adjusted		
Variables	B	*p*‐value	St.B*	95% CI	*p*‐value
*Moderate intensity PA*					
Age over/under 12 years	−27.8	**<0.001**	−0.3	−39.0 to −6.0	**0.009**
Sex	−4.0	0.486	−0.0	−16.3 to 8.4	0.519
Father's BMI	0.1	0.855			
Mother's BMI	−1.3	**0.046**	−0.0	−2.7 to 2.2	0.837
Father's education	9.2	0.152			
Mother's education	4.7	0.423			
Participating in organized sports	−16.6	**0.024**	−0.5	−49.3 to −3.3	**0.023**
Screen time per day	−5.7	**0.017**	0.1	−8.9 to 12.3	0.738
Total sleep time	0.0	**0.002**	−0.4	−0.0 to 0.0	0.092
Midpoint sleep	−0.0	**<0.001**	−0.5	−0.0 to 0.0	0.057
*Vigorous intensity PA*					
Age over/under 12 years	−22.9	**<0.001**	−0.4	−27.5 to −0.9	**0.038**
Sex	−8.4	**0.048**	−0.2	−19.9 to 7.1	0.338
Father's BMI	−0.1	0.855			
Mother's BMI	−0.8	0.070	−0.1	−2.0 to 1.3	0.681
Father's education	4.1	0.404			
Mother's education	0.2	0.964			
Participating in organized sports	−11.8	**0.040**	−0.4	−30.0 to 0.6	0.059
Screen time per day	−3.7	**0.043**	0.3	−2.0 to 12.3	0.150
Total sleep time	0.0	**<0.001**	−0.2	−0.0 to 0.0	0.389
Midpoint sleep	−0.0	**<0.001**	−0.5	−0.0 to −0.0	**0.019**

*Note*: Statistical significant *p*‐values < 0.05 are presented in bold.

Abbreviations: BMI, body mass index; PA, physical activity; St.B*=standardised Beta.

More time in MPA was related to age and participation in organized sports. Children ≤12 years (CI: −39.0 to −6.0, *p* = 0.009) and individuals who participated in organized sports (CI: −49.3 to −3.3, *p* = 0.023) were most likely to spend time in MPA. A tendency toward an association between earlier sleep timing and higher MPA was also indicated, but this was not statistically significant (CI: −0.0 to 0.0, *p* = 0.057).

More time in VPA was related to age and sleep timing. Children ≤12 years (CI: −27.5 to −0.9, *p* = 0.038) and individuals with earlier sleep timing (CI: −0.0 to −0.0, *p* = 0.019) were most likely to spend more time in VPA. There was also a tendency toward an association between participation in organized sports and higher VPA, though not statistically significant (CI: 30.0–0.6, *p* = 0.059).

## 4 DISCUSSION

The main findings of the study were that children and adolescents with severe obesity spent less time in moderate and vigorous physical activity compared to normal weight peers, while there was no difference for time spent in light physical activity or sedentary activity between the groups. Higher physical activity level in the group of children with severe obesity was associated with lower age (≤12 years), participation in sports and earlier sleep timing.

In line with most previous studies, the group of children with severe obesity spent significantly less time in moderate and vigorous physical activity than their normal weight peers.^35^, ^36^, ^37^, ^38^ In the current study, children and adolescents with severe obesity and children and adolescents with normal weight spent 72 min/day and 105 min/day in MVPA, respectively, somewhat more time than demonstrated in most comparable studies.^9^ This might indicate that Norwegian children in general are more physically active than children in many other countries, due to less unsafe areas, more outdoor leisure time facilities and more government funded organized sports. Another factor explaining these differences could be that wrist worn accelerometers, as used in the current study, is found to be more sensitive to physical activity in low active children than hip‐worn accelerometers used in several other studies.^9^, ^27^

In the current study, mean physical activity levels in both children with severe obesity and normal weight are in line with international guidelines that recommend 60 min of daily MVPA for children and adolescents.^3^ This is contrary to a systematic review stating that children and adolescents with obesity did not achieve the recommended minimum amount of 60 min daily MVPA in most of the included studies.^9^ However, only 60.7% of the children with severe obesity in the current study met the recommendations despite a group mean above 60 min MVPA per day underlining the heterogeneity of the group with regard to physical activity level.

Differences in MVPA related to age were also observed in the present study. Children (≤12 years) spent less time in sedentary and LPA, and more time in MPA and VPA compared to adolescents, regardless of weight status. Steene‐Johannessen et al.^39^ reported that 87% of Norwegian six‐year‐old girls and 94% of the boys met the recommendations of 60 min daily MVPA, meanwhile only 40% of the fifteen‐year‐old girls and 51% of the fifteen‐year‐old boys did. The results from the current study showed a similar decrease in physical activity between children and adolescents (Figure 1), in both individuals with severe obesity and with normal weights, indicating that other factors might be of more importance than weight status in explaining this trend. Plausible explanatory factors for the decrease in PA by age could be changes in daily activities, including school hours, homework, travel distance to school, and a change in pleasure activities toward more sedentary activities.^40^

As expected, and observed in previous studies, children with severe obesity below the age of 12 had more MPA and VPA than children over the age of 12.^41^ Further, MPA time was strongly associated with participation in organized sports. In general, it appears that sport participation contributes to higher levels of MVPA in children and adolescents of normal weight, but in children with obesity the findings are inconclusive.^42^ Only 31.8% of the children and adolescents with obesity in the current study engaged in organized sports. A previous review of qualitative studies has reported that children with severe obesity may experience more barriers to and less support for participation in organized sports than their normal weight peers.^5^ A review of physical activity interventions for childhood obesity also concluded that close supervision and support during the activities predicted better weight related outcomes.^43^ Even though active play is found to be at least as effective as organized sports in accumulating MVPA in children,^44^ it might seem that the children not participating in organized sports also engage less in spontaneous active play.^43^ Thus, the MVPA does not seem to be as easily obtained through active play or self‐organized activities compared to organized sports for these children with severe obesity. Further, the strongest association discovered for time spent in VPA in the current study was with sleep timing, where earlier sleep timing was related to increased VPA during the day. This was shown regardless of total sleep time, proposing that early sleep timing have a closer association to time spent in VPA than the total amount of sleep. These results mirror a previous study presenting that despite of having similar total sleep time, children with late sleep timing engaged in less pedometer‐measured MVPA compared to a group of children with early sleep timing.^22^ The causality of the relationship between earlier sleep timing and more physical activity could not be established in this cross‐sectional study, but it is likely that a higher level of physical activity during the day may promote an earlier sleep timing.

Screen time, gender, parental BMI and sleep duration were not factors associated to time spent in physical activity in the current study. Previous studies have reported mixed results for screen time use and sleep duration and activity levels.^16^, ^17^ while being female^24^ and having parents with obesity^45^ are associated with decreased activity levels. In the current study screen time was not differentiated (e.g., gaming, social media use, watching TV). As most children and adolescents has a quite extensive screen time it might not differentiate between adolescents with different activity levels. Some data, however, indicate that specific screen behaviors, such as gaming, is more strongly related to later sleep timing,^46^ and possibly to less MVPA, but this needs to be examined in further studies.

Assessing physical activity and sleep with objective measures over seven consecutive days, is an advantage of the present study as well as high compliance, low percentage of missing data and a matched comparison group. Registration of physical activity was performed using ActiWatch wrist‐worn accelerometer, while in other studies hip‐worn accelerometers have most often been used.^35^, ^47^ ActiWatch was preferred in the current study because of its ability to measure both physical activity and sleep. However, in the study of Ekblom et al.,^27^ ActiWatch was reported to have a tendency to register lower levels of physical activity in highly active children and higher levels in low‐active children compared to the hip‐worn ActiGraph.^27^ This could influence the total amount of physical activity reported in the low‐active children. Comparison of ActiWatch placed on hip and wrist show that total measured activity was lower on the hip than on the wrist,^48^ which could be relevant for the interpretation of accelerometer data in the present study. However, the cut‐offs used for the different intensities of physical activity were developed for wrist‐worn accelerometers. Despite the differences in count calculations, evidence still prefers the wrist placement because it is likely to increase compliance due to the easy way of wearing the device.^30^, ^48^ Also, to be noted, different cut‐off limits for physical activity‐intensities between studies hampers the comparison of activity levels. However, a cross‐validation of wrist‐worn and hip‐worn accelerometers, found that the two devices gave similar estimates of time spent in MVPA at group level, but should not be compared on an individual level.^27^, ^49^

Regarding sleep measures, sleep diaries were not used as a support for the objective measures, presenting a limitation of the study. Due to the cross‐sectional design of this study conclusions regarding causal relationships cannot be made, and there is always a possibility of residual confounding in observational studies. For physical activity measures, only physical activity registered in wake time between 8 AM–9 PM were included in the analysis. This could represent a limitation, as the children might engage in physical activity at other times. However, this definition of time frame is in line with previous used scoring protocols for objectively measured physical activity.^27^ Parent reported data represents a limitation due to the possibility of reporting bias. Also, screen time was measured using one question only and there was no differentiation between types of screen activities. A differentiation could have increased the value of this information.

This study demonstrates, as hypothesized, that treatment seeking children and adolescents with severe obesity engage in less physical activities than their normal weight peers. Only 1/3 of the children with severe obesity were engaged in organized sports and these children demonstrated increased physical activity level. Organized sports might therefore be particularly important for children with obesity to achieve more health promoting physical activity. Qualitative studies exploring barriers to engaging in organized physical activity for children with obesity reported barriers such as lack of family and peer support, perceptions of being negatively judged by others or not experiencing competence compared to others when participating in sports.^5^ Fatigue and physical discomfort, as well as dislike of being “visible”, and problems with finding appropriate clothing for sports were also reported as barriers.^5^ This knowledge might guide clinicians or sports coaches on how to support children with obesity in organized sports for example, adults taking the responsibility for choosing teams, demonstrating a zero tolerance for negative comments or providing team clothing in all sizes. In treatment of childhood obesity providing a setting for organized physical activity with peers of similar weight status might be a good idea. Further, to encourage families to participate in sports where a larger body size and muscular strength is an advantage in order to enhance sports related self‐efficacy (e.g., American football or downhill skiing). In the current study late sleep timing emerges as a factor related to lower physical activity level. Other studies have also found sleep timing to be related to more screen time and higher intake of energy dense food.^50^, ^51^ Addressing sleep habits might be a good starting point for lifestyle intervention, as it might be easier to change than food and activity habits and might create more energy to engage in physical activity.

## 5 CONCLUSION

Children and adolescents with severe obesity were less physically active than normal weight peers. Higher levels of physical activity for the group of children with obesity were associated to lower age (≤12 years), participation in organized sports and earlier sleep timing. The present findings highlights the need for considering both sleep timing and participation in organized sports as possible dimensions in treatment of childhood obesity. In clinical practice, this could enhance the effectiveness of interventions targeting childhood obesity.

## AUTHOR CONTRIBUTIONS

Hanna F. Skjåkødegård, Yngvild S. Danielsen and Petur B. Juliusson conceived and designed the study. Hanna F. Skjåkødegård, Yngvild S. Danielsen, Malin Kleppe and Petur B. Juliusson collected and scored the data. Sven J.G. Olsson developed the tailor‐made software for processing physical activity data. Yngvild S. Danielsen, Marit Mongstad, Bente Frisk and Sigurd W. Hystad performed statistical analyses. Yngvild S. Danielsen and Hanna F. Skjåkødegård wrote the paper in consultation with Marit Mongstad, Sigurd W. Hystad, Sven J.G. Olsson, Malin Kleppe, BF and Petur B. Juliusson. All authors discussed the results and contributed to the final manuscript.

## CONFLICT OF INTEREST

The authors declares no conflict of interest.
